# Formation of a Volunteer Harmful Algal Bloom Network in British Columbia, Canada, Following an Outbreak of Diarrhetic Shellfish Poisoning

**DOI:** 10.3390/md11114144

**Published:** 2013-10-29

**Authors:** Lorraine McIntyre, David Cassis, Nicola Haigh

**Affiliations:** 1 British Columbia Centre for Disease Control, 655 West 12th Avenue, Vancouver, BC, V5Z 4R4, Canada; 2 AquaBC Consulting, 2610 West 10th Avenue, Vancouver, BC, V6K 2J7, Canada; E-Mail: davidcassis@gmail.com; 3 Harmful Algae Monitoring Program, Centre for Shellfish Research, Vancouver Island University, 900 Fifth Street, Nanaimo, BC, V9R 5S5, Canada; E-Mail: nicky.haigh@viu.ca

**Keywords:** DSP, biotoxin, monitoring network, public health, phytoplankton, surveillance

## Abstract

Evidence for shellfish toxin illness in British Columbia (BC) on the west coast of Canada can be traced back to 1793. For over two hundred years, domestically acquired bivalve shellfish toxin illnesses in BC were solely ascribed to paralytic shellfish poisonings caused by algal blooms of *Alexandrium*. This changed in 2011, when BC experienced its first outbreak of diarrhetic shellfish poisoning (DSP). As a result of this outbreak, Canada’s first DSP symposium was held in November, 2012, in North Vancouver, BC. Three of the objectives of the symposium were to provide a forum to educate key stakeholders on this emerging issue, to identify research and surveillance priorities and to create a DSP network. The purpose of this paper is to review what is known about shellfish poisoning in BC and to describe a novel volunteer network that arose following the symposium. The newly formed network was designed for industry shellfish growers to identify harmful algae bloom events, so that they may take actions to mitigate the effects of harmful blooms on shellfish morbidity. The network will also inform public health and regulatory stakeholders of potentially emerging issues in shellfish growing areas.

## 1. Introduction

Marine shellfish are an important industry in the province of British Columbia (BC), Canada, contributing over $233 million annually to the economy [1]. Commonly consumed and harvested shellfish in BC (aquaculture or wild caught) include oysters, clams, mussels, scallops, geoducks, cockles (bivalves) and crabs, shrimps, sea cucumbers, sea urchins and prawns [1]. However, marine shellfish may cause shellfish poisoning and may themselves be adversely affected by their environment and dietary food source, phytoplankton, leading to slow growth and shellfish mortality. Only 60 to 80 phytoplankton species, mostly from algal groups, such as dinoflagellates and diatoms, are considered harmful out of the estimated 4000 known species [2]. High accumulations of phytoplankton species that produce toxins are referred to as harmful algal blooms (HABs). In patches of very high abundance, phytoplankton blooms may produce harmful effects, including lethal reductions in dissolved oxygen, as well as toxins that can be accumulated by shellfish. These blooms may be responsible for human illnesses and deaths, as well as fish, shellfish and marine animal mortalities [2]. Phytoplankton’s ability to produce toxins depends on genetic and environmental factors [2,3]. For example, Bricelj *et al.* (2005) found that genetic mutations conferring greater resistance to paralytic shellfish poisoning (PSP) toxins to shellfish with the mutation allow more PSP toxins to be absorbed by these shellfish and may drive ecosystem changes towards PSP toxin-resistant shellfish with the potential to cause harmful effects to shellfish consumers [4].

This paper will review shellfish illnesses in BC, marine biotoxin and phytoplankton monitoring and will describe the formation of a volunteer, industry-led phytoplankton monitoring network.

### 1.1. Shellfish Poisoning

Shellfish poisoning can occur through two routes: intoxication or infection. Toxin-mediated shellfish poisoning occurs when toxin-containing phytoplankton or bacteria are ingested by shellfish. Infectious shellfish poisoning is caused by bacteria, parasites or virus present in shellfish. Bivalve shellfish are filter-feeders, naturally ingesting phytoplankton, bacteria, parasites and viruses from the surrounding water. Ingested microorganisms and associated biotoxins become concentrated in the tissues of bivalve shellfish [5]. The toxins that are present may be harmful to shellfish consumers, as well as to shellfish. Marine biotoxins of concern in Canada for human illness include those that cause PSP, amnesic shellfish poisoning (ASP), diarrhetic shellfish poisoning (DSP) and seafood toxin illnesses of an undetermined nature [6]. Elsewhere in the world, neurotoxic and azaspiracid shellfish and ciguatera fish poisonings occur, but these intoxications have not been linked to fish or shellfish products harvested in Canadian marine waters [2,3,7]. Rarely reported cases of ciguatera intoxication in BC are linked to imported fish products. Domestic shellfish sources in BC have to date been limited to PSP and DSP illnesses only.

### 1.2. History of Shellfish Poisonings in British Columbia

BC’s first outbreak of DSP occurred in July, 2011, when sixty-two illnesses were attributed to mussels consumed from 15 different retail locations [8]. Marine biotoxins detected in mussel samples taken from the implicated harvest area included okadaic acid (OA) and dinophysis toxins (DTX-1 and DTX-3), indicating that this cluster of illnesses was due to DSP [8]. It is likely these toxins were produced by *Dinophysis acuminata*, as it was present in 82% of the samples taken 25 km from the mussel harvest area [9].

PSP cases have been documented in Eastern Canada since the 1880s and, in BC, from nearly 100 years earlier [6,10]. Shellfish poisoning, likely PSP, was described in 1793, when one of Captain George Vancouver’s crew died and four others became ill after consuming a meal of mussels in Poison Cove, on BC’s central coast [10]. The illnesses were likely caused by the accumulation of toxins in the mussels, based on reported symptoms and the fact that the mussels were roasted before they were eaten. PSP has been a reportable condition in BC since 2001, although case reports are relatively rare. An estimated 47 illnesses (no deaths) from 20 separate incidents were documented from various sources between 1990 and 2012 [11].

The first known outbreak of amnesic shellfish poisoning (ASP) occurred in eastern Canada in 1987 with cultured mussels, causing over 100 illnesses, including hospitalizations and three deaths [6,12]. Although the associated toxin, domoic acid, remains a concern in BC and shellfish monitoring for domoic acid has been in place since 1988, there have been no reported illnesses of ASP in this province [6]. Shellfish closures due to elevated domoic acid in BC are also infrequent; only twice in the last ten years have levels above 20 µg/g been detected in indicator species, most recently in Haida Gwaii (Queen Charlotte Islands) in 2012 [13].

Infectious shellfish poisonings in BC include those caused by *Vibrio* species, bacterial organisms prevalent in marine waters, and norovirus, an environmentally-resistant virus, with humans as the only known reservoir [14,15]. Most incidents attributed to bivalve shellfish consumption in BC result from these two agents. with the majority of illnesses attributed to *V. parahaemolyticus.* Average annual case counts of *V. parahaemolyticus* were 20.3, with a case rate in BC of 0.5 per 100,000 population (between 2001 and 2006); rarely cases are attributed to *V. cholerae* non-01/0139, *V. fluvialis* and *V. hollisae* [16]. Norovirus has been linked to at least two outbreaks in BC, while the most recent outbreak in 2010 was likely caused by harvesters who were themselves ill; the outbreak in 2004 was traced to shellfish harvested from multiple locations in BC coastal waters, with no point source contamination identified [17,18].

### 1.3. Impact of Harmful Algal Blooms on the Shellfish Industry

In addition to the effects of marine biotoxins on human health, HABs may also be a problem for shellfish growers in BC through the lethal and sublethal effects on the shellfish. Several species of dinoflagellates (*Protoceratium reticulatum*, *Noctiluca scintillans*, *Cochlodinium fulvescens*), as well as raphidophytes (*Heterosigma akashiwo*), silicoflagellates (*Dictyocha fibula* and *D. speculum*) and diatoms (*Rhizosolenia setigera*, large *Chaetoceros* species) can produce irritating chemicals, cause mechanical damage and prevent normal feeding or create low oxygen conditions that may generate shellfish seed mortalities and periods of low growth in larger shellfish [19,20,21,22]. Other species present in BC (*Myrionecta rubra*, *Rhizosolenia chunii*, cryptophytes) can render shellfish unmarketable, as they can cause transient changes in the quality of the product, such as a bitter taste or red colouring [23].

The damages and economic issues produced by harmful algae for the United States (USA) shellfish industry have been estimated between 30 and in excess of 80 million dollars annually [24,25]. The main costs of HABs have been separated into four categories: (1) public health issues and the cost of caring for those affected; (2) commercial impacts that include the delays in harvest due to biotoxin contamination and changes in product quality by nuisance species, reduced production due to losses of seed and adult shellfish caused by mortalities and loss of consumer confidence in shellfish aquaculture products; (3) tourism and recreation impacts, such as closed recreational fisheries due to biotoxins, as well as nuisance effects on beaches (foams, odours); and (4) monitoring and management costs. According to these estimates, public health costs account for the largest portion of the annual economic impact of HABs, closely followed by the commercial costs, with both types of damages covering up to 80% of the annual impact.

### 1.4. Shellfish Marine Biotoxin and Phytoplankton Monitoring Programs

Shellfish monitoring programs in Canada are run by three government agencies, the Canadian Food Inspection Agency (CFIA), the Department of Fisheries and Oceans Canada (DFO) and Environment Canada, who collaborate to deliver the Canadian Shellfish Sanitation Program [26]. In BC, all bivalve shellfish are required to be processed in federally registered facilities, which only accept shellfish that have been harvested in an approved area and tag them for commercial distribution, such that they are traceable from producer to consumer. One of CFIA’s roles is the provision of marine biotoxin monitoring: based on 2012 data, CFIA conducted over 10,000 tests for PSP/ASP and 1100 for DSP to verify that shellfish from BC are safe [13,26].

Marine biotoxin monitoring programs for shellfish can be successfully supplemented with phytoplankton monitoring for early detection of HABs. These programs exist in many other countries, such as Chile, Denmark, France, Japan, New Zealand, Norway and Spain, and in several local, state-wide and national programs in the USA, although the structure and oversight of these programs vary [27,28,29]. One nation-wide example in the USA is the National Oceanic and Atmospheric Administration (NOAA) funded Phytoplankton Monitoring Network (PMN), which has explored innovative approaches to interacting with volunteers, such as the creation of the Phyto phytoplankton identification application for mobile devices [30]. In Scotland, the Scottish Association for Marine Science (SAMS), on behalf of the Food Standards Agency Scotland, has obtained weekly samples at 36 sites throughout the summer since 2005 and also requests shellfish farms to submit voluntary samples for analysis [31]. Privately funded phytoplankton monitoring can be found mostly related to the aquaculture industry. In Chile, the Instituto Tecnológico del Salmón has conducted phytoplankton monitoring through Plancton Andino S.A. since 2002 [32].

In Canada, DFO manages phytoplankton monitoring programs that began in 1987 on the Atlantic coast, coinciding with the ASP outbreak, but programs have declined in the years following [28,33]. Weekly phytoplankton monitoring occurs at the Institut Maurice Lamontagne for the St. Lawrence estuary (Quebec, Canada) and at the St. Andrews Biological Station for the Bay of Fundy (New Brunswick). There are no government funded phytoplankton monitoring programs (for either fish or shellfish) on the Pacific coast of Canada.

In contrast to Canada’s lack of HAB monitoring on the west coast, in Washington State, south of BC, extensive HAB monitoring is conducted through the SoundToxin project led by the Northwest Fisheries Science Centre, NOAA. NOAA is a federal agency (within the USA Department of Commerce, which maintains multiple laboratories across the USA, many of which operate in collaboration with one or more educational institutions). Under the Olympic Region Harmful Algal Bloom Partnership, HAB monitoring results are shared with management agencies, coastal Indian tribes, businesses, academic institutions and public interest groups [34].

In BC, phytoplankton monitoring is done in association with the salmon aquaculture industry. The Harmful Algae Monitoring Program (HAMP) was formed in 1999 and is currently based at Vancouver Island University in Nanaimo. This program annually monitors up to 27 coastal sites to assist salmon farmers with monitoring, management and mitigations of HABs [28]. HAMP is able to provide real-time warning of harmful species, such as *Heterosigma akashiwo*, the most common cause of salmon mortality in BC [35]. Data generated by the program has been compiled in a database that may be used in the future to aid in HAB prediction. HAMP has had a great deal of success in educating fish farm technicians to routinely analyze samples at their own sites. Farmers are trained in microscopic phytoplankton identification and supplied with the HAMP Harmful Plankton Handbook, which is updated annually [36].

### 1.5. The Journey from a DSP Outbreak to a Volunteer Phytoplankton Monitoring Network

The 2011 DSP outbreak highlighted our knowledge gaps about marine biotoxin issues in general, including DSP as an emergent issue with shellfish in BC. Many questions were raised by public health monitoring agencies and the industry during this period, such as, “Why did this outbreak occur?”, “What, if anything, had changed in the marine environment to introduce or establish this new marine biotoxin threat in the Pacific Northwest?”, “How could we improve our management, either through improved monitoring, improved disease surveillance or better risk communication?” and “What collaborative opportunities could be identified between agencies and industry to foster improved management?” These questions formed the basis of the Canadian DSP symposium, held in November, 2012. The objectives and learning goals, planned to be inclusive for all stakeholders, were: (1) to provide a forum to educate key stakeholders on this emerging issue; (2) to create a DSP network; (3) to identify research and surveillance priorities in BC; (4) to build capacity in BC to respond to DSP and other shellfish toxin outbreak investigations; and (5) to optimize risk communication to stakeholders and the public during outbreaks and harmful algal bloom events. This paper will describe the research and surveillance priorities identified at the symposium, as well as the collaborative opportunities resulting from this symposium that led to the formation of BC’s first volunteer harmful algal bloom network for shellfish growers.

## 2. Methods

**Figure 1:**
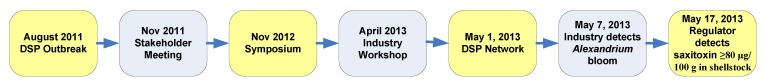
Events leading to the formation of the volunteer phytoplankton monitoring network. DSP, diarrhetic shellfish poisoning.

The idea to hold a symposium about DSP was first introduced at a shellfish risk reduction meeting in November 2011, attended by various public health, regulatory and industry groups. Start-up funding was secured, and planning for the symposium began in the spring of 2012. The Canadian DSP symposium was held on 27 November 2012 (see the timeline in Figure 1). One important outcome was the formation of an industry-driven volunteer phytoplankton monitoring network that arose following a workshop based on presentations made at the symposium.

### 2.1. Symposium Structure for Garnering Feedback

A working group for the symposium included representatives from the shellfish industry association, BC Shellfish Grower’s Association (BCSGA), two federal agencies (Health Canada and CFIA) and provincial representation by BCCDC. All provided monetary and in-kind funding. There was no registration fee in order to encourage a diversity of participants. Participants were solicited via e-mail to networks of organizing members and places held for specific stakeholder groups. Funding facilitated registration of 111 participants (including speakers) and reimbursement of speaker and venue expenses. Opportunities for feedback were provided before, during and after the symposium, and each participant received a package with an evaluation form containing the symposium objectives and five questions. Questions asked whether the symposium identified research and collaboration opportunities and asked participants to further identify steps and describe opportunities for improving knowledge, communications and furthering research collaborations.

### 2.2. Establishment of a Volunteer Harmful Algal Monitoring Network for Shellfish Growers in BC

One session of the DSP symposium was devoted to phytoplankton monitoring. Industry members, including BCSGA, were receptive to presentations from the US and from Canada highlighting opportunities for HAB monitoring. Following the symposium, the BCSGA incorporated a HAB monitoring workshop into their annual meeting. Presenters at the symposium, Nicky Haigh and David Cassis, instructed the BCSGA-sponsored workshop on Phytoplankton Monitoring and Information Networking on 18 April 2013 in Lund, BC, Canada. Attendees received information on the ecology of plankton, plankton sampling, microscopes and sample analysis, harmful algae species description and observation, using plankton monitoring for shellfish site husbandry improvement, networking and online resources. Practical demonstrations for plankton sampling and microscopy were also included. Following these activities, a brief sum-up and discussion ensued in which a Google group was proposed and accepted at the workshop’s conclusion. This listserv-based network was initially formed with interested workshop participants, as well as representatives of government agencies involved in environmental and biotoxin monitoring (CFIA, DFO, Environment Canada and BCCDC) and academics from the University of British Columbia and Vancouver Island University.

## 3. Results

### 3.1. Symposium Evaluation

**Figure 2:**
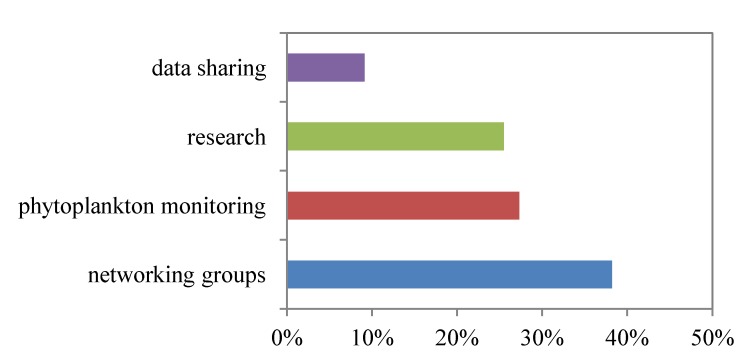
Symposium participant feedback describing needs for improved shellfish management.

The response rate to the symposium evaluation was 63% (*n* = 70). The majority of symposium participants (62%) self-identified as belonging to the public health/regulator/inspector group, followed by laboratory (20%), industry (14%) and university/other (4%) groups. The feedback received overwhelmingly supported the needs for improved collaboration and phytoplankton monitoring (Figure 2). Fifty-five comments articulated next steps for improving marine toxin knowledge, research and collaborative opportunities [13]. The need for networking groups to improve collaboration was the most frequently expressed (38%) in comments, such as: “greater/increased collaboration with Washington State research”, “on-line/collaboration/info-sharing site” and “collaboration between DFO-CFIA.” Twenty-seven percent of respondents identified a need for “phytoplankton monitoring for the shellfish industry as an aid in preventing shellfish poisoning outbreaks”. Requests to focus on other research areas (25%) and data sharing (9%) were also noted: “get DFO science back into HAB research”, “economic value of industry and impact of recall to industry”, “convergence of information from all parties, including CSSP, WDOH, HAMP, industry groups, *etc.*” and “include actual data for both HABs and toxins in BC”.

When asked what participants learned from the symposium, half demonstrated gaining either basic (14%) or advanced (36%) knowledge of the topics discussed, demonstrating the diversity of background and acquired knowledge in the audience (Table 1). They learned about phytoplankton monitoring and its value (20%), followed by knowledge about program roles for shellfish management, laboratory testing, USA collaboration and research and knowledge gaps. Most participants (83%) either agreed or strongly agreed that the symposium identified opportunities for networking, research and collaboration. However, when asked if the symposium identified communications between government, industry and researchers, over one third either disagreed or were undecided [13].

**Table 1:** Summary of responses to DSP evaluation survey.

Responses to evaluation survey	*n* (%)
Q. 2 Identify things learned (*n* = 106)	
Advanced knowledge	36 (34.0)
Phytoplankton monitoring	20 (31.1)
Basic knowledge	14 (13.2)
Program roles for shellfish management	13 (12.2)
Laboratory testing	12 (11.3)
US collaborations and research	6 (5.7)
Knowledge gaps	5 (4.7)

Following the symposium, participant contact information, links, speaker presentations and other information were shared on the BCCDC DSP symposium web page. A presentation about the symposium was also given to the Canadian Institute of Public Health Inspectors on 8 May 2013 [37]. Industry support for a spring 2013 workshop led to the formation of the volunteer harmful algal bloom network described below.

### 3.2. Formation and Structure of the Volunteer Harmful Algal Monitoring Network in BC

**Figure 3:**
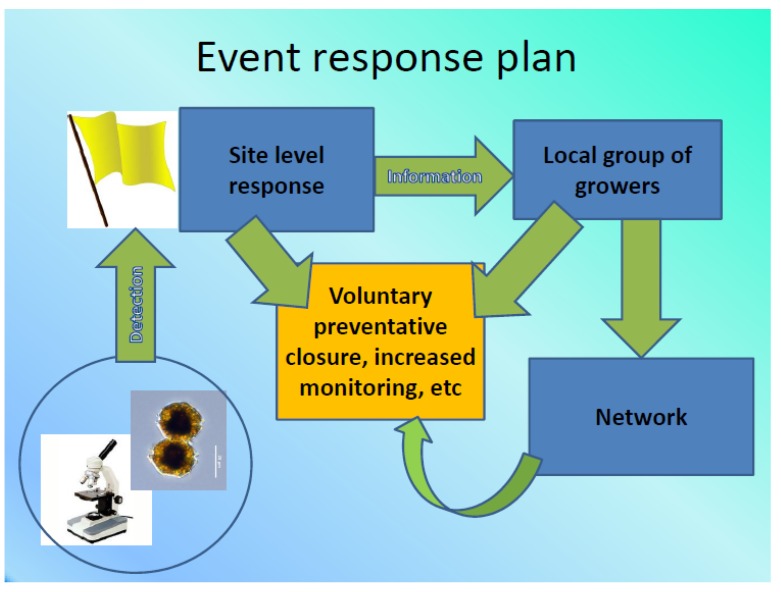
Algal identification event response plan overview for shellfish industry network participants.

The purpose of forming a volunteer network was to address the existing knowledge gap presented by the lack of phytoplankton monitoring on BC’s coast. Active monitoring can inform the shellfish industry if harmful algae are present in their harvest areas, so they may avoid situations leading to spat death, to costly food recalls or to the sale of product leading to consumer illness. The volunteer network was established following a shellfish industry workshop attended by 30 shellfish growers (April, 2013). Harmful algae species-specific event response protocols indicate possible actions and precautions for shellfish growers. These protocols were created specifically for the workshop and were also distributed to network members. An overview of the algal identification event response plan presented to those attending the workshop is depicted in Figure 3 and examples of recommended resources shown in Table 2. When to report, what species to report and how reports should be made were articulated in the event response protocols developed for shellfish growers in BC. The protocol involves three levels of response: farm level, local area level and network level. Response protocols vary depending on the type of species detected during monitoring. For example, any detections of *Alexandrium* would result in enhanced vigilance at the farm, including harvest moratoriums. Nearby growers should also be alerted, and information on the event report sheet should be recorded and distributed to the network. Other species, such as rare detections of *Pseudo-nitzschia,* undergo assessment of ASP alerts reported on Canadian and US government monitoring sites. If no reports exist on these sites, the detection is recorded, but not distributed, although increased vigilance (monitoring at the farm level) is still required. The kinds of data recommended for reporting at the farm level include photographic evidence of microscopic algae identifications, observations of water (color and appearance) and of animals (behaviour and mortalities) and seawater measurements (salinity, temperature).

**Table 2:** Educational resources provided to shellfish growers at the workshop to establish proficiency in phytoplankton monitoring and identification.

Resource	Source
Plankton Identification Handbook for the Shellfish Growers on the West Coast of Canada	[38]
Harmful Algae Bloom Event Response Protocols for the Shellfish Grower of British Columbia 2013	[39]
Phyto’ pedia—The Phytoplankton Encyclopaedia Project	[40]
List of On-line Resources Provided by BC Shellfish Grower’s Association	[41]

Resources and materials necessary to begin phytoplankton monitoring were shared with members of the Google group [41], and listed on the BCSGA website on 1 May 2013 [41]. The network membership is managed through this Google group and currently includes industry members, as well as members from academia and government agencies. Network members now use the Google group to report the occurrence of harmful algae, their location and abundance, as well as to share digital images for confirmation by experts in phytoplankton identification. The information contained in these reports can then be used by other members of the group to take action and precautions that are appropriate to the threats identified.

Since the network was established, an early report of *Alexandrium* was noted by one shellfish grower on 7 May 2013. As indicated in the event response protocols, *Alexandrium* was treated as a possible contamination, and the shellfish growers involved communicated the results to the network and coordinated efforts to increase local phytoplankton monitoring. Agencies involved in toxin monitoring (CFIA) received the information and confirmed the presence of saxitoxin in shellfish samples above allowable limits on 17 May 2013, resulting in area closures near this site [42]. The participation of representatives of government agencies in this network is helpful for increasing the dialog with shellfish growers. This exchange of information in real time helps the government agencies to conduct a more flexible responsive toxin monitoring that can address particular concerns before more serious problems arise.

## 4. Discussion and Conclusions

While the Canadian shellfish sanitation program is extensive and testing of shellfish at sentinel sites for marine toxins is usually done weekly, not all sites are tested that often. There is the potential for HABs to develop in under a week and for contaminated shellfish to reach the marketplace. The evidence demonstrates that public health and the shellfish industry should be concerned**.** Over half (55%) of the 20 PSP incidents involving illness that were described between 1990 and 2012 were traced to commercially harvested shellfish purchased at retail outlets [11]. Recalls of shellfish products also reveal a similar trend—a review of the online CFIA recall database found 17 marine biotoxin recalls in 2011–2012: four involved public warnings for PSP and DSP, and 13 were directed to commercial retailers. The majority of the recalls (80%) concerned products from BC waters, with three from Quebec and New Brunswick.

Toxin concentrations in marine animal tissues can vary greatly depending on the phytoplankton present, as well as seasonal, geographical, harvesting and processing factors [5,7]. HABs are influenced by temperature spikes, the introduction of nutrients and fresh water during spring freshets and changing thermoclines, which have all contributed to the well-recognized global increase in HABs [2,5,7,43]. Detection of harmful algae in plankton is challenging, because harmful algae species may be present without causing negative effects. However, detection can be a trigger to raise the level of preparedness and proceed with cautionary actions.

With no phytoplankton monitoring in place for shellfish interests in the years leading up to the 2011 outbreak, there was no knowledge among the shellfish industry or regulatory monitoring agencies of the occurrence of potentially harmful algal species. Several weeks prior to the 2011 outbreak, *Dinophysis* counts of up to 24,000 cells L^–^^1^ were detected by staff who routinely monitor harmful algal blooms for aquaculture fish farms [9]. If a communications network had existed between the fish and shellfish industry and regulatory stakeholders or if phytoplankton monitoring had existed for shellfish growers, it is possible that the DSP outbreak in 2011 could have been avoided. Shellfish growers, through routine algae monitoring of their sites, can now identify harmful algae, empowering them to safely grow and harvest product for commercial sale and avoid costly recalls. The formation of the volunteer industry network will also forewarn monitoring agencies and public health when HABs are present. Feedback received from participants at the Canadian DSP symposium recommended collaborations between regulatory authorities in BC, with industry and with USA agencies. The need for phytoplankton monitoring, improved responsiveness to HABs and a focus on research are requirements for the continuing health of the shellfish industry. Recent recalls of shellfish highlight how rapidly HABs develop and how commercially harvested shellfish pose a risk for marine biotoxin acquired illnesses in domestically harvested shellfish, even with the excellent biotoxin monitoring program in place for shellfish on the west coast of Canada.

The establishment of the newly formed industry-funded volunteer harmful algae network reinforces the importance of holding multi-stakeholder research meetings to further the aims of industry and public health. We recommend government involvement in the network and that greater attention be paid to the development of resources on the west coast of Canada, specifically that the federal government focus be directed towards HAB research in western Canada and that partnerships are formed with USA colleagues sharing this coastline and these concerns [43]. Existing infrastructures in eastern Canada could be used as a model to establish and improve phytoplankton monitoring on the west coast. Our ability to provide an early warning system to forecast and predict harmful algae blooms will benefit the economic health of the industry and will also protect the public health of our communities.
